# Luspatercept: A New Tool for the Treatment of Anemia Related to β-Thalassemia, Myelodysplastic Syndromes and Primary Myelofibrosis

**DOI:** 10.3390/diseases10040085

**Published:** 2022-10-09

**Authors:** Eleftheria Hatzimichael, Despoina Timotheatou, Epameinondas Koumpis, Leonidas Benetatos, Alexandros Makis

**Affiliations:** 1 Department of Haematology, Faculty of Medicine, School of Health Sciences, University of Ioannina, 45500 Ioannina, Greece; 2 Medical Department, Genesis Pharma SA, Halandri, 15232 Athens, Greece; 3 Blood Bank, Preveza General Hospital, 48100 Preveza, Greece; 4 Department of Child Health, Faculty of Medicine, School of Health Sciences, University of Ioannina, 45500 Ioannina, Greece

**Keywords:** luspatercept, anemia, beta-thalassemia, myelofibrosis, myelodysplastic syndromes, ineffective erythropoiesis, TGF-beta inhibitor

## Abstract

Anemia is a common feature of both benign and malignant hematologic diseases. Beta-thalassemia (β-thalassemia) syndromes are a group of hereditary disorders characterized by ineffective erythropoiesis, due to a genetic deficiency in the synthesis of the beta chains of hemoglobin, often accompanied by severe anemia and the need for red blood cell (RBC) transfusions. Myelodysplastic syndromes (MDS) are characterized by cytopenia(s) and ineffective hematopoiesis, despite a hypercellular bone marrow. Primary myelofibrosis (PMF) is a clonal myeloproliferative neoplasm characterized by reactive fibrosis of the bone marrow, accompanied by extramedullary hematopoiesis. Luspatercept, previously known as ACE-536, is a fusion protein that combines a modified activin receptor IIB (ActRIIB), a member of the transforming growth factor-β (TGF-β) superfamily, with the Fc domain of human immunoglobulin G (IgG1). It has shown efficacy in the treatment of anemia due to beta β-thalassemia, MDS and PMF and recently gained approval by the Federal Drug Agency (FDA) and the European Medicines Agency (EMA) for transfusion-dependent (TD) patients with β-thalassemia and very low to intermediate-risk patients with MDS with ringed sideroblasts who have failed to respond to, or are ineligible for, an erythropoiesis-stimulating agent. In this review, we describe the key pathways involved in normal hematopoiesis and the possible mechanism of action of luspatercept, present its development and data from the most recent clinical trials in β-thalassemia, MDS and PMF, and discuss its potential use in the treatment of these hematological disorders.

## 1. Introduction

Luspatercept, previously known as ACE-536, is a modified activin receptor type IIB ligand trap (ActRIIB) which is currently being tested for use in diseases characterized by ineffective erythropoiesis, such as beta-thalassemia (β-thalassemia), myelodysplastic syndromes (MDS) and primary myelofibrosis (PMF) [1,2,3]. Clinical development of luspatercept was initiated after the incidental observation that another ligand trap, sotatercept, an activin receptor type IIA, was associated with an increase in the levels of hemoglobin (Hb) in postmenopausal women when administered for osteoporosis [4]. In preclinical studies, luspatercept has been shown to enhance late-stage erythroid maturation, by inhibiting the GDF11-mediated Smad2/3 signaling, both in a steady state and under conditions of stress, regardless of the level of erythropoietin (EPO) [5,6]. Following initial findings, phase I, II and III clinical trials were designed and launched in healthy volunteers [6], β-thalassemia [1,7] and MDS [8], and in PMF with or without a Janus kinase (JAK) inhibitor (NCT03194542, NCT04717414). In this review, we describe the key pathways involved in normal hematopoiesis and the possible mechanism of action of luspatercept, present its development and data from the most recent clinical trials in β-thalassemia, MDS and PMF, and discuss its potential use in the treatment of these hematological disorders.

### 1.1. Key Pathways in Erythropoiesis

A regulatory network including various cytokines, mainly EPO and regulators of iron metabolism, are involved in erythropoiesis, which can adjust to changing physiological needs and to various pathological conditions. Erythropoiesis is tightly regulated by a complex network of transcription factors, among which GATA-binding protein 1 (GATA-1), the master transcriptional regulator of erythropoiesis, is essential for differentiation, maturation and globin chain synthesis. GATA-1 is protected in the nucleus during terminal erythroid differentiation by heat shock protein 70 (HSP70), a supervisor protein that enables the refolding of proteins denatured by cytoplasmic stress to prevent their aggregation [9]. 

The process of erythropoiesis is also regulated by members of the transforming growth factor-beta (TGF-β) superfamily. This superfamily contains more than thirty soluble proteins, such as the bone morphogenetic proteins (BMPs), activins and growth differentiation factors (GDFs) [10]. 

**Figure 1:**
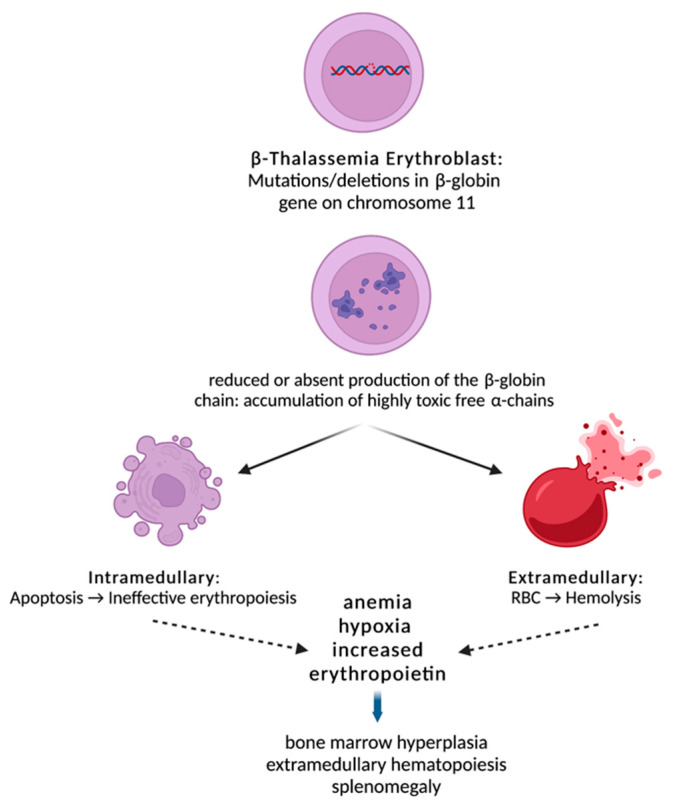
Beta-thalassemia is characterized by reduced or absent production of the β-globin chain due to deletions or mutations in the β-globin gene. This results in an α/β-chain imbalance and accumulation of highly toxic free α-chains leading to intramedullary apoptosis and peripheral hemolysis. Ineffective erythropoiesis in β-thalassemia leads to anemia and, subsequently, to tissue hypoxia and overexpression of EPO, which results in accumulation of early erythroid progenitors. However, the erythroid progenitor maturation process fails, and erythroblasts succumb to apoptosis. Over time, the combination of tissue hypoxia, increased EPO and ineffective erythropoiesis creates a vicious cycle that ultimately leads to a massive expansion of erythroblasts.

BMPs and GDFs interact with combinations of type I and type II receptor dimers, such as activin receptors II A and B (ActRIIA and ActRIIB), to produce multiple possible signaling complexes, leading to the activation of one of two competing sets of SMAD transcription factors [11]. GDF11 binds to both ActRIIA and ActRIIB and phosphorylates Smad and non-Smad intracellular signaling proteins [12]. SMADs are responsible for the downstream signal transduction to the nucleus (Figure 1). GDF11 is mainly expressed in early, immature erythroid progenitors and is necessary for their survival and for inhibition of terminal differentiation [13].

### 1.2. Ineffective Erythropoiesis in β-Thalassemia 

Beta-thalassemia is an inherited monogenic disorder characterized by reduced or absent production of the β-globin chain, resulting in an α/β-chain imbalance and accumulation of highly toxic free α-chains [14]. Ineffective erythropoiesis in β-thalassemia leads to anemia and, subsequently, to tissue hypoxia and overexpression of EPO, which results in accumulation of early erythroid progenitors. However, the erythroid progenitor maturation process fails, and erythroblasts succumb to apoptosis [9]. Over time, the combination of tissue hypoxia, increased EPO and ineffective erythropoiesis creates a vicious cycle that ultimately leads to a massive expansion of erythroblasts (Figure 1) [15]. In parallel, α-globin chains isolate HSP70 in the cytoplasm, resulting in cleavage of GATA-1 by caspase 3. This results in end-stage maturation arrest and apoptosis at the erythroblast stage [9]. As noted above, GDF11-ActRIIB-Smad2/3-dependent signaling is a key regulatory mechanism in proliferating erythroid precursors, controlling their late-stage maturation both in a steady state and under conditions of stress. 

### 1.3. Ineffective Erythropoiesis in MDS

MDS are a heterogeneous group of clonal hematopoietic disorders characterized by ineffective hematopoiesis, bone marrow failure and dysplasia in hematopoietic cells of one or more cell lineages, with a varying risk of transformation to acute myeloid leukemia (AML). Low-risk MDS usually present with anemia, carry a low risk of transformation to AML and are associated with prolonged overall survival (OS) [16]. Anemia in lower-risk MDS is managed symptomatically with RBC transfusions and/or EPO-stimulating agents (ESAs), in particular, recombinant epoetin-alfa [8] and beta [17]. About one-third of patients, however, exhibit primary resistance to ESAs, or lose response over time, following two years of treatment.

In MDS, overactivation in the TGF-β signaling has been described [18]. In addition, higher levels of expression of GDF11 were noted in low-risk MDS patients compared to healthy controls [19], while GDF11 levels were negatively correlated with hemoglobin levels in low-risk MDS patients and with late-stage erythroblasts and RBC levels in high-risk MDS patients [20]. Therefore, TGF-β-directed suppressive treatments could restore late-stage erythropoiesis in MDS patients and ameliorate their anemia.

### 1.4. Ineffective Erythropoiesis in PMF 

PMF is a BCR-ABL1-negative myeloproliferative neoplasm (MPN) characterized by anemia, abnormal cytokine expression leading to reticulin fibrosis and constitutional symptoms, extramedullary hematopoiesis hepatosplenomegaly, leukemic progression and shortened survival [21,22]. 

The molecular mechanisms that govern PMF pathophysiology include driver mutations (JAK2, c-MPL, calreticulin) and sub-clonal mutations in genes involved in epigenetic regulation, RNA splicing, protein ubiquitination and apoptosis. Apart from the pathways affected by these mutations, other deregulated pathways include the PI3K/AKT/mTOR, the hedgehog and the TGF-β pathways. In particular, TGF-β1 is highly expressed in PMF and is involved in the pathogenesis of fibrosis and megakaryocyte hyperplasia [23,24]. In addition, GATA1 transcription factor expression is reduced in PMF megakaryocytes, which is thought to contribute directly to PMF pathogenesis under the negative control of TGF-β1 [25,26].

Currently, the only treatment that can prolong survival or provide a potential cure for patients with PMF is allogeneic stem cell transplant (AlloSCT), which is associated with significant treatment-related mortality and severe morbidity. Alternative treatment options are mainly palliative, with no modification of the natural history of the disease. Hydroxyurea may be used in the proliferative phase of PMF and has been shown to be effective in reducing the splenic size by half in approximately 40% of patients [27]. Ruxolitinib and fedratinib are JAK inhibitors that have shown spleen volume reduction and amelioration of constitutional symptoms. In MF, anemia stems from multiple factors that are mutually related and only partially understood. Besides constitutive activation of the JAK–STAT pathway and dysregulated inflammatory cytokine production, leading to inhibition of bone marrow erythropoiesis, sequestration and destruction of circulating erythrocytes by the enlarged spleen is one of the factors contributing to the pathogenesis of anemia in MF patients. Disease-related anemia can be exacerbated by treatment with ruxolitinib and to a lesser extent with fedratinib because of myelosuppression [28,29]. The limited treatment options for PMF-associated anemia include androgens, recombinant erythropoietin [30], prednisone and thalidomide, but they offer low response rates and short duration of response [22]. Momelotinib is a small-molecule JAK1/JAK2 inhibitor that suppresses hepcidin expression, leading to stimulation of erythropoiesis [31]. Clinical data from the phase 2 and the two phase 3 SIMPLIFY trials showed high rates of sustained transfusion independence [32,33]. In the Momentum trial, momelotinib will be tested against danazol, seeking registrational approval [34]. Iron chelation has also been studied in PMF patients and two Italian multicenter studies recently reported iron chelation and erythroid response [35,36]. Pre-clinical models suggest that TGF-β1 inhibition might have a beneficial effect in reactivating normal hematopoiesis in PMF [3,37]. Thus, the current data provide a rationale for exploring the efficacy of agents acting against the TGF-β1/GDF11-ActRIIA/B-SMAD2/3 pathway in promoting erythropoiesis. 

## 2. The Mode of Action of Luspatercept: Ligand Traps of Activin Receptor II in Ineffective Erythropoiesis 

**Figure 2:**
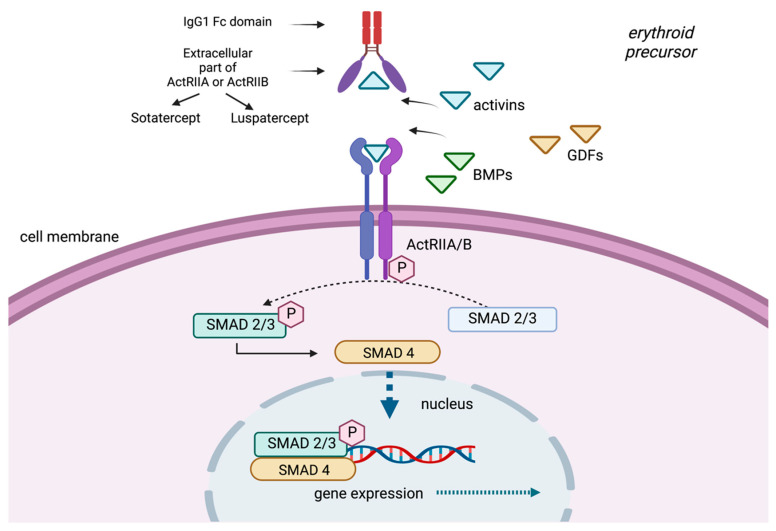
The TGF-β signaling pathway and its ligand traps.

Sotatercept (ACE-011) is a chimeric protein consisting of an extracellular domain of ActRIIA and the Fc domain of human immunoglobulin G1 (IgG1) (Figure 2). Early preclinical studies evaluated its murine analogue, RAP-011, as it was thought that targeting activin receptor signaling, and possibly other TGF-β superfamily members, could be of therapeutic value in osteoporosis-related bone loss [38]. Unexpectedly, sotatercept demonstrated a notable erythroid response when administered to otherwise healthy subjects with osteoporosis, with the aim of increasing bone density. This observation was not reported as an adverse event, but on the contrary, the authors identified that this effect could be beneficial to patients with ineffective erythropoiesis [39]. Nevertheless, due to this finding, the drug was not further explored in the setting of postmenopausal osteoporosis.

Luspatercept (ACE-536) is a fusion protein consisting of a modified human activin IIB receptor (ActRIIB) extracellular domain, with the Fc domain of human immunoglobulin G1 (IgG1). Although it may appear similar to sotatercept, their potential to trap activin A differs [16]. It has been proposed that luspatercept binds GDF11 and inhibits GDF11-ActRIIB-Smad2/3-dependent signaling, promoting late-stage maturation of erythroid precursors [19]. However, a recent preclinical study by Guerra et al. showed the lack of GDF11 did not improve anemia or prevent the activity of RAP-536 in a mouse model of β-thalassemia, indicating that GDF11 is not the only target of luspatercept [40].

TGF-β ligands, activins, GDFs and BMPs bind to a type II receptor (ActRIIA, ActRIIB), which phosphorylates receptor-regulated Sma and Mad-related proteins (R-SMAD). R-SMAD/coSMAD complexes gather in the nucleus, where they enable transcription and participate in the regulation of target gene expression. Sotatercept and luspatercept are modified receptors of activin (activin receptor-II trap ligands) that have been developed from the fusion of the extracellular part of activin receptors (ActRIIA or ActRIIB) and the Fc part of IgG immunoglobulin. They function as a selective trap ligand that inhibits the activation of the SMAD pathway.

## 3. Clinical Development of Luspatercept

### 3.1. Phase I Clinical Trials in Healthy Volunteers

The first in-human, phase I clinical trial of luspatercept was conducted in 32 healthy postmenopausal women with a mean age of 59 years who were randomized in sequential cohorts of eight subjects to receive up to two doses of either 0.0625, 0.125 and 0.25 mg/kg or a placebo (3:1 randomization), given subcutaneously (sc) every two weeks [6]. Luspatercept was well tolerated at dose levels of up to 0.25 mg/kg over the one-month treatment period, and the incidence of adverse events (AEs) was comparable across treatment groups, including the placebo. Most AEs (83%) were mild in severity and were considered unrelated to the study treatment. The Hb increased from baseline 7 days after initiation of treatment with luspatercept. The mean increase was dose-dependent and was maintained for several weeks following treatment. The proportion of subjects with Hb increase from baseline ≥1.0 g/dL increased in a dose-dependent manner to 83.3% of subjects in the highest dose group of 0.25 mg/kg [6].

### 3.2. Clinical Development of Luspatercept in β-Thalassemia

Two phase II single-arm, multicenter studies, specifically a dose-finding base and an open-label extension study (NCT01749540 and NCT02268409), were conducted to determine the tolerable and recommended dose and schedule for adult patients with TD or non-TD (NTD) β-thalassemia [7]. The primary endpoint was erythroid response, defined as an increase in Hb from baseline of ≥1.5 g/dL for ≥2 weeks (in the absence of RBC transfusions) for NTD patients, and a reduction in RBC transfusion burden over a 12-week period of ≥20%, compared with pre-treatment for TD patients. In the three-month dose-finding study, 35 patients were treated with six escalating dose levels from 0.2 to 1.25 mg/kg of luspatercept every three weeks. In the extension cohort, doses from 0.8 to 1.25 mg/kg were administered to 29 patients. The primary endpoint was met, as protocol-defined erythroid response occurred in 71% of NTD patients and 81% of TD patients receiving the higher doses of luspatercept. Pharmacokinetic analysis confirmed that 1.0 mg/kg of luspatercept is an appropriate starting dose for further studies in patients with β-thalassemia, with titration up to 1.25 mg/kg. The use of luspatercept at a dose of 0.6–1.25 mg/kg led to a mean increase in Hb of ≥1.5 g/dL from baseline for at least 14 days in more than half (58%) of the NTD patients. Of the TD patients, most (81%) recorded a ≥20% reduction in RBC transfusion requirements [7].

Luspatercept was generally safe and well tolerated in all 64 patients treated, at all dose levels, with most AEs being grade one or two, mainly during the first eight weeks, with a clear trend to decrease with time. The most frequent AEs (≥10%) were bone pain, headache, myalgia, arthralgia, musculoskeletal pain, back pain and injection site pain. No treatment-related grade-four AEs or serious AEs or treatment-related deaths were reported, and only 4/64 (6%) patients discontinued treatment because of AEs [41].

The BEYOND trial is a phase II, double-blind, randomized, placebo-controlled, multicenter study to determine the efficacy and safety of luspatercept vs. placebo in adults with NTD β-thalassemia (NCT03342404). The primary objective is to evaluate the effect of luspatercept versus placebo on anemia, as measured by increase in mean Hb in the absence of transfusions over a continuous 12-week interval, from week 13 to week 24, compared to baseline. The study has completed recruitment and results were recently presented [42]. It included 145 patients, of which 96 were treated with luspatercept, while 49 received a placebo. A total of 74 (77.1%) patients treated with luspatercept achieved the primary endpoint versus none out of 49 in the placebo arm (*p* < 0.0001). The primary endpoint was achieved by 40 of 55 (72.7%) luspatercept patients with mean baseline Hb< 8.5 g/dL (versus 0 in placebo arm; *p* < 0.0001), and 34 of 41 (82.9%) with mean baseline Hb ≥ 8.5 g/dL (versus 0 in placebo arm; *p* < 0.0001) [42]. Moreover, in the same study, investigators found that luspatercept not only reduced RBC transfusion burden in patients with NTD β-thalassemia, but also improved quality of life [43]. Regarding the adverse events, no thromboembolic events occurred in either arm [42]. 

The BELIEVE study (NCT02604433), a phase III, double-blind, randomized, placebo-controlled, multicenter study, was designed to determine the efficacy and safety of luspatercept plus BSC versus placebo plus BSC in TD adults with β-thalassemia [1]. The inclusion criteria included TD adult patients with β-thalassemia or Hb E/β-thalassemia. Patients were randomized 2:1 to receive either luspatercept or a placebo every three weeks for ≥48 weeks [1]. Patients in both treatment arms continued to receive RBC transfusions and iron chelation therapy to maintain the same baseline Hb level. The primary endpoint was a ≥33% reduction in RBC transfusion burden, with a reduction of ≥2 RBC units, during weeks 13 to 24. Key secondary endpoints included ≥33% reduction in transfusion burden and at least 2 RBC units in the later time period, at weeks 37 to 48, and ≥50% reduction in transfusion burden and at least 2 RBC units at weeks 13 to 24, and at the later time period, at weeks 37 to 48, and mean change in RBC transfusion burden at weeks 13 to 24. A total of 336 patients were randomized with a median age of 30 years (range 18–66 years). The β^0^/β^0^ genotype was observed in 30% patients in the luspatercept arm and 31% patients in the placebo arm. The primary endpoint was met by a significantly greater proportion of patients in the luspatercept arm: 21.4% (48/224) of patients vs. 4.5% (5/112) of patients in the placebo arm achieved a reduction of at least 33% in the RBC transfusion burden, and of ≥2 RBC units, during weeks 13 to 24 [1].

All the subgroups benefited consistently from luspatercept treatment, even the difficult-to-treat patients, such as those with a β^0^/β^0^ genotype or patients receiving more than 6 RBC units/12 weeks at baseline. The more demanding endpoint of a ≥50% reduction in RBC transfusion burden plus ≥ 2 RBC at weeks 13–24 and 37–48 was achieved by 7% and 10% of the 224 patients receiving luspatercept, respectively, compared to 1.8% and 0.9% of 112 patients receiving a placebo, and the difference was statistically significant. In addition, 40.2% of patients receiving luspatercept achieved a ≥50% reduction in transfusion burden over any consecutive 12 weeks of the study, compared to 6.3% patients on placebo, while for any 24-week period, the percentages were 16.5% and 0.9%, respectively. For the patients that achieved a reduction in the transfusion burden of at least 33% and 50%, a reduction of approximately 6 and 8 RBC units, respectively, was estimated per patient per 24 weeks. Response was observed earlier for non-β^0^/β^0^ than for β^0^/β^0^ genotype patients, according to a post hoc analysis. The median longest duration of response with luspatercept was around 100 days for those who responded during any 12-week interval [1]. 

Concerning iron parameters, in the limited duration of 48 weeks of the study, the least square mean difference in serum ferritin levels was −348 μg per liter (95% CI, −517 to −179) between treatment arms. This was not clinically relevant for liver iron concentration and myocardial iron deposition, probably due to the chronic nature of the disease. Iron accumulation is a slowly reversible procedure and this reduction in serum ferritin levels, together with the indirect benefit of the reduction of the transfusion burden and the continuing chelation therapy, mark a positive trend in the iron balance.

Treatment-emergent AEs (TEAEs) observed in the BELIEVE study were generally consistent with data already reported from phase II studies. TEAEs of all grades were reported more frequently in the luspatercept treatment, including bone pain (19.7% vs. 8.3%), arthralgia (19.3% vs. 11.9%), dizziness (11.2% vs. 4.6%), hypertension (8.1% vs. 2.8%) and hyperuricemia (7.2% vs. 0%). Bone pain was more frequent during the first 12 weeks in both arms; it was rather of short duration and could be alleviated with regular analgesia. No malignancy or pre-malignant indication was reported. Thromboembolic events occurred in eight patients (3.6%) in the luspatercept arm and in one patient (0.9%) in the placebo arm. All the eight patients in the luspatercept group had undergone splenectomy and had at least one risk factor for thromboembolic events. The discontinuation rate due to an adverse event was 5.4% for the luspatercept arm and 0.9% for the placebo, and no deaths were reported in either treatment group [1].

Luspatercept thus demonstrated significant reductions in the RBC transfusion burden in adults with TD β-thalassemia and was granted FDA and EMA approval for patients with β-thalassemia who require regular RBC transfusions. A phase 2a study (NCT04143724) evaluating the safety and pharmacokinetics of luspatercept in regularly transfused pediatric patients due to β-thalassemia is ongoing.

Luspatercept thus demonstrated significant reductions in RBC transfusion burden in adults with TD β-thalassemia and was granted FDA and EMA approval for patients with β-thalassemia who require regular RBC transfusions. A Phase 2a study (NCT04143724) evaluating the safety and pharmacokinetics of luspatercept in regularly transfused pediatric patients due to β-thalassemia is ongoing.

The role of luspatercept on extramedullary hematopoiesis needs to be further explored, since the data are limited and conflicting. There is one case of recurrent spinal cord compression due to an extramedullary hematopoietic mass (EHM) in a thalassemic patient on luspatercept that was ultimately treated with radiotherapy [44]; one case of a TD patient that achieved transfusion independence with a later asymptomatic increase of the EHM and another case with a >50% reduction in the transfusion burden and stable EHM [45]. As the authors suggest, luspatercept might be a new therapeutic option for these patients, by improving ineffective erythropoiesis and decreasing the need for transfusions; on the other hand, patients need to be monitored closely and discontinue treatment if they do not respond in order to avoid undesired erythroid expansion [45].

### 3.3. Clinical Development of Luspatercept in MDS

PACE-MDS (NCT01749514) was a phase II, multicenter, open-label, dose-finding study for adult patients with lower-risk MDS or non-proliferative chronic myelomonocytic leukemia (CMML) with anemia, with or without the need for RBC transfusion support [46]. All patients were to receive luspatercept every 21 days at dosages ranging from 0.125 mg/kg to 1.75 mg/kg, for a maximum of 12 weeks; then, after assessment for safety and efficacy, they could be considered for enrolment in the extension study (NCT02268383). In the extension cohort, patients received 1.0 mg/kg, with dose titration up to 1.75 mg/kg, for a maximum of five years. Due to the short duration of treatment, a modified endpoint was introduced as the primary endpoint in the 12-week base study, namely the proportion of patients achieving modified International Working Group (IWG)-defined hematological improvement–erythroid (HI-E), defined as an increase in Hb ≥ 1.5 g/dl from baseline for 14 days or longer in low transfusion burden (LTB) patients and a reduction in RBC transfusion requirements of ≥4 units or more, or a 50% or higher reduction in RBC units over eight weeks compared with pre-treatment transfusion burden in high transfusion burden (HTB) patients [47]. Following review of the efficacy data, dosages of 0.125–0.5 mg/kg were deemed sub-therapeutic, and only patients treated at higher dose concentrations (51/58) were included in the efficacy evaluable population. Of the 51 patients treated with higher luspatercept dose concentrations (0.75–1.75 mg/kg), across both base and extension studies, 32 (63%) patients achieved IWG HI-E. Of the 17 LTB patients, 65% (11) achieved HI-E, and 13 showed sustained increases in mean Hb from baseline for at least 15 months, of which 11 (85%) maintained the Hb increase for a median duration of 8.3 months. Of the HTB patients, 62% (21/34) treated with higher doses (0.75–1.75 mg/kg) achieved HI-E, while 79% (15/19) achieved IWG HI-E with a median response duration of 11.6 months. Achievement of HI-E appeared to be similar, regardless of prior use of ESAs or lenalidomide. According to the baseline serum level of EPO, 76% of patients with EPO < 200 IU/L, 58% of patients with EPO ≥ 200 and ≤500 IU/L, and 43% of patients with EPO > 500 IU/L achieved HI-E; thus, luspatercept appeared effective, even in patients with higher EPO, where ESAs show poor activity. Luspatercept treatment produced more frequent and more robust responses in patients with RS ≥ 15% (69% achieved IWG HI-E) or SF3B1 mutations (77% achieved IWG HI-E). Regarding RBC transfusion independence (RBC-TI), 38% of evaluable patients (16/42) achieved RBC-TI across both studies, most of whom had previously failed ESA treatment. Of the previously transfused patients who continued receiving luspatercept into the extension study, 50% (11/22) remained transfusion-free for ≥8 weeks, with a median duration of RBC-TI of 15.3 months. Multivariable logistic regression analysis showed that EPO (<100 IU/L vs. ≥100 IU/L; *p* = 0.04) and SF3B1 mutation status (yes vs. no; *p* = 0.01) had a significant effect on IWG HI-E. EPO (<500 IU/L vs. ≥500 IU/L; *p* = 0.02), and iron chelation therapy use (yes vs. no; *p* = 0.01) exerted significant effects on RBC-TI. Analysis of safety showed that grade-three AEs considered related to treatment were reported in only 5% of patients who received at least one dose of the study drug. In summary, the PACE-MDS study reported that 1.0 mg/kg luspatercept with up to 1.75 mg/kg titration is the appropriate starting dose for further studies in patients with MDS, that the drug is effective in patients with high endogenous EPO, regardless of prior ESA use, and that response was more frequent and robust in patients with RS ≥ 15% or SF3B1 mutations [46]. 

Following the PACE-MDS study, a phase III, double-blind, randomized, placebo-controlled, multicenter study was designed to evaluate the efficacy and safety of luspatercept vs. placebo in subjects with anemia due to very low, low or intermediate MDS with RS who required RBC transfusions, named the MEDALIST study (NCT02631070) [48]. Eligible patients were adults with MDS of very low, low or intermediate risk according to the revised international prognostic scoring system (IPSS-R), with RS according to the WHO 2016 criteria, who were refractory, intolerant or unlikely to respond to ESAs and required regular RBC transfusions (≥2 units/8 weeks in the 16 weeks prior to randomization). The patients were randomized 2:1 to receive luspatercept, at a starting dose of 1.0 mg/kg with dose escalation up to 1.75 mg/kg or placebo, every three weeks for ≥24 weeks, with no cross-over allowed. The primary endpoint was the proportion of patients who were RBC-TI over any consecutive eight weeks or longer within week 1 through week 24. The key secondary end point was the proportion of patients who were RBC-TI over any consecutive 12-week period or longer within week 1 through 24 and week 1 through 48. A total of 229 patients (63% male) were randomized and received luspatercept (*n* = 153) or a placebo (*n* = 76). The median age was 71 years and median time from diagnosis was 41.8 months; 218 (95%) patients had previously received ESAs and 213/218 (98%) had disease refractory to ESAs. The primary endpoint of RBC-TI for eight weeks or longer was met by 58/153 patients (38%) receiving luspatercept, compared with 10/76 (13%) patients receiving a placebo, and this difference was statistically significant (*p* < 0.001). All subgroups consistently benefited from luspatercept treatment, but patients with a RBC transfusion burden < 4 units/8 weeks during the 16 weeks prior to treatment benefited the most. Regarding the key secondary endpoint, 43 patients (28%) receiving luspatercept achieved RBC-TI for 12 weeks or longer, compared to six (8%) in the placebo arm during the first 24 weeks. The median duration of the longest single period of RBC-TI in primary endpoint responders was 30.6 weeks for luspatercept and 13.6 weeks for placebo. A mean increase of at least 1.0 g/dL was achieved in 35% in the luspatercept arm vs. 8% in the placebo arm during weeks 1 through 24, and 41% vs. 11% during weeks 1 through 48, respectively. The safety profile was consistent with that reported in the phase II PACE-MDS study [46]. AEs of any grade occurring in ≥10% of patients were fatigue (27% vs. 13%), diarrhea (22% vs. 9%), asthenia (20% vs. 12%), nausea (20% vs. 8%), dizziness (20% vs. 5%) and back pain (19% vs. 7%) in the luspatercept vs. the placebo arm, respectively. Grade-three or four AEs occurred in similar percentages in the luspatercept and placebo arm (42% vs. 45%) [48]. 

Longer-term efficacy analyses of the MEDALIST study showed that 45.1% of patients treated with luspatercept and 15.8% treated with placebo achieved RBC-TI ≥ 8 weeks. In addition, during weeks 1–48, RBC-TI ≥ 16 weeks was achieved by 28.1% of patients treated with luspatercept and by 6.6% of patients that received a placebo (*p* < 0.0001). AEs were more common in luspatercept than in the placebo arm and they included fatigue and diarrhea [49]. Regarding other secondary endpoints of the MEDALIST study, neutrophil and platelet counts were increased with luspatercept in lower-risk MDS [50]. Of the 25 patients evaluable for HI-Neutrophil, more luspatercept vs. placebo-treated patients achieved HI-Neutrophil during weeks 1 to 24 (13.3% vs. 0.0%) and weeks 1 to 48 (20.0% vs. 10.0%). Of the 14 patients evaluable for HI-Platelet, more luspatercept vs. placebo-treated patients achieved HI-Platelet during weeks 1 to 24 (50.0% vs. 33.3%) and weeks 1 to 48 (62.5% vs. 33.3%). It is worth noting that the number of evaluable patients was very low, and the results should be cautiously assessed. Paradoxically, infections, mainly grade-one or two, were more frequent in patients treated with luspatercept vs. placebo (53.6% and 40.8%) [50]. Furthermore, in the MEDALIST trial, the health-related quality-of-life outcomes were without meaningful changes within groups and between groups across all domains [51]. Patients treated with luspatercept remained longer on treatment and had more durable and repeated episodes of RBC-TI response events than patients treated with a placebo. The median time from randomization to AML progression for patients treated with luspatercept was 61.7 months (range 56.7–223.6) vs. 32.7 months (range 30.1–60.4) with the placebo [52]. Moreover, patients randomized to luspatercept in the MEDALIST trial had an increase of erythropoiesis-associated biomarkers (e.g., blood reticulocyte counts, ratio of reticulocytes/soluble transferrin receptor 1) [53]. A retrospective analysis from the MEDALIST trial showed that 23 participants had Myelodysplastic Syndrome/Myeloproliferative Neoplasm with Ring Sideroblasts and Thrombocytosis (MDS/MPN-RS-T). Out of these participants, 14 were treated with luspatercept, while nine received a placebo. The primary endpoint of RBC-TI for ≥8 weeks during weeks 1–24 was achieved by 64.3% of patients treated with luspatercept vs. 22.2% of patients who received a placebo (odds ratio 11.3; 95% confidence interval [CI] 1.19, 106.12; *p* = 0.028) [54].

The COMMANDS study (NCT03682536), a phase III, open-label, randomized study, was also designed to compare the efficacy and safety of luspatercept with that of epoetin-alpha for the treatment of anemia in ESA naive patients with IPSS-R very low, low or intermediate-risk MDS who require RBC transfusions. The presence of RS is not an eligibility criterion. The primary endpoint of the study is RBC-TI over the first 24 weeks. Secondary endpoints are HI-E per IWG within 24 weeks, time to and duration of HI-E, duration of RBC-TI ≥ 24 weeks and ≥84 days, and evaluation of changes in QoL, pharmacokinetic factors, and time to AML and OS.

A retrospective study of patients with MDS and RS treated with luspatercept was recently published [55]. This study included 39 patients. Thirty-two patients (87%) were IPSS-R very low/low-risk patients at the time of initial diagnosis and thirty-one patients were transfusion-dependent at the time of treatment initiation. Overall, 5/31 (16%) transfusion-dependent patients and 2/8 of transfusion-independent patients (25%) met the criterion of anemia response [55]. Multivariable analysis of variables identified Epo level > 80 IU/L at diagnosis and ALC ≥ 1.8 × 10^9^/L at time of luspatercept treatment initiation as predictors of response. Median response duration was six months for transfusion-dependent patients, while the duration of response for the two transfusion-independent patients was three and seven months, comparable to the eight months observed in the MEDALIST trial. Of note, among the total seven responding patients, four lost their response in 3–7 months. The small size of the responding population and the transient nature of response made the authors question the cost-effectiveness of the drug [55]. This discrepancy highlights the need for more real-world data, not only in MDS, but also in thalassemic patients, since clinical trials have specific eligibility criteria and a certain design, and their results may not be applicable to a more general population.

Whether luspatercept can substitute ESAs in low-risk MDS patients is being studied in the COMMANDS trial, a randomized, phase III trial enrolling approximately 350 patients with LR-MDS who require at least 2 U RBC transfusion every eight weeks as an upfront strategy (NCT03682536). What would be really interesting to test is the combination of luspatercept and ESAs in low-risk MDS patients, since the former affects late-stage hematopoiesis and the later early stage.

### 3.4. Clinical Trials of Luspatercept in PMF

Given the association between luspatercept inhibiting factors in PMF pathogenesis, and the pre-clinical data on luspatercept in β-thalassemia and MDS, studies in PMF are warranted. At the time of writing, no full-paper results evaluating luspatercept in PMF were available, but two ongoing studies are registered on clinicaltrials.gov. The first is a phase II, multicenter, open-label study design evaluating the efficacy of luspatercept in PMF, post-polycythemia vera MF and post-essential thrombocythemia MF-associated anemia in TD and NTD patients (NCT03194542). All patients received luspatercept at 1 mg/kg sc on day 1 of a 21-day cycle, initially for 169 days, and responders continued for an additional 1.5 years, and the primary endpoint is an increase in Hb of 1.5 g/dL from baseline values over any consecutive 84-day period without RBC transfusion in the RBC-TI, while TD patients should become RBC-TI over any consecutive 84-day period. A total of 74 patients with MF were enrolled. Cohort 1 included 41 patients who did not receive ruxolitinib and did not receive any RBC transfusion; in Cohort 2, patients did not receive ruxolitinib but had received 2–4 RBC transfusions per month in the 12-week period before enrollment; 33 enrolled patients received a stable dose of ruxolitinib for at least 16 weeks, of which 14 did not receive RBC transfusions (Cohort 3A) and 19 were transfusion dependent (Cohort 3B). An absolute Hb increase ≥ 1.5 g/dL at every measurement from baseline over any consecutive 12 weeks was noted in 2/20 (10%) and 3/14 (21%) patients in Cohorts 1 and 3A, respectively. RBC-TI over any consecutive 12 weeks was achieved by 2/21 (10%) and 6/19 (32%) patients in Cohorts 2 and 3B. Notably, median duration of Hb response was 20 weeks in Cohort 1 and 12 weeks in Cohort 3A. A minority of AEs were grade 3–4 in severity, and no treatment-related deaths were noted [56].

The second currently recruiting study (NCT04064060) is a phase IIIb, open-label, single-arm, rollover trial that addresses long-term safety in a large number of patients (estimated enrollment: 665) with MDS, β-thalassemia, PMF and MPN-associated MF who have participated in other luspatercept clinical trials. The patients will receive 1 mg/kg luspatercept on the first day of a 21-day cycle. Primary endpoints include occurrence of AEs, progression to high/very high-risk MDS or AML and occurrence of other malignancies, and the secondary outcome includes overall survival. 

## 4. Practical Considerations for Use of Luspatercept in Clinical Practice

Luspatercept is administered subcutaneously at a starting dose of 1 mg/kg every 21 days for both MDS with RS and TD-thalassemic patients, and the dose can be further adjusted based on response [1]. A full blood count should be taken prior to each administration in order to assess the hemoglobin levels [1]. Further experience is needed to determine the dose and the frequency of administration after the first year of treatment. In the BELIEVE trial, there were early and late responders, in 12–24 week intervals and in 37–48 week intervals, respectively. Response was observed earlier for non-β^0^/β^0^ than for β^0^/β^0^ genotype patients [1]. 

Regarding the adverse events, as mentioned above, thromboembolic events occurred in eight splenectomized patients in the luspatercept arm versus one patient in the placebo arm. Considering that thalassemia is a hypercoagulable state, thromboembolic events are expected [57]. However, the prescribing information of luspatercept suggests monitoring patients for signs and symptoms of thromboembolic events and institute treatment promptly. Thromboprophylaxis should also be considered in high-risk patients for thromboembolic events. Blood pressure should also be measured before each administration. Luspatercept is not an appropriate treatment for women considering pregnancy, since it may cause fetal harm, while breastfeeding is also not recommended (SPC). 

## 5. Other Indications and Possible Future Uses of Luspatercept

Except for β-thalassemia and MDS with RS, luspatercept and other activin receptor ligand traps seem to be promising treatments for Diamond–Blackfan anemia and other types of anemia [58,59]. Finally, luspatercept may also have synergistic or additive action when used with other pharmacological agents, such as recombinant erythropoietin, or other agents targeting the TGF-β pathway, such as Galunisertib [59,60,61].

## 6. Conclusions

Luspatercept, a fusion protein consisting of ActRIIB, a modified activin receptor IIB and a member of the TGF-β superfamily, and IgG1, the Fc domain of human immunoglobulin G, has been studied in the treatment of β-thalassemia and lower-risk MDS and MF following the serendipitous discovery of the drug’s effect on erythropoiesis. Based on the BELIEVE and MEDALIST trial data, respectively, luspatercept has recently been approved by the FDA and EMA for the treatment of TD β-thalassemia patients and for patients with very low to intermediate-risk MDS with RS who have failed or are ineligible for an EPO-stimulating agent.

The precise mode of action of luspatercept has not been completely elucidated. Gene profiling is not a routine process for patients with β-thalassemia and no specific mutations or markers have yet been identified to predict response. In thalassemic patients, luspatercept is well tolerated and its benefits outweigh the risks of gene therapy, as age and performance status are not a barrier. It could at any time ‘bridge’ gene therapy in the case of no or partial response. The reduction in RBC transfusion burden is of great importance, since it is associated with infections and complications related to iron overload (e.g., cardiac failure) [62,63]. Moreover, the reduction in RBC transfusion leads to a decrease in clinic visits and, subsequently, improvement in quality of life. In low-risk MDS, the treatment options are very limited when patients do not respond or lose response to ESAs and their needs for RBC transfusions increase. The dosing scheme of luspatercept promotes compliance; however, more data are needed regarding the duration of response. Results from clinical trials in MDS patients will answer the question whether luspatercept is more efficacious than or can be combined with ESAs. Regarding PMF-associated anemia, the initial results from the ongoing phase II study suggest significant clinical activity of luspatercept in these setting, including those receiving concomitant ruxolitinib. A minority of AEs were grade 3–4 in severity, consistent with previous studies in MDS and beta-thalassemia. Real-world data are also eagerly awaited to confirm the clinical trial data and further define the safety profile of the drug. Finally, since ineffective erythropoiesis is a feature of various conditions, the use of luspatercept should be further explored in order to be established as a new tool for treating anemia besides thalassemia and MDS with RS.
